# Targeting hypoxia-inducible factor 1α in chronic graft-versus-host disease to enhance graft-versus-leukemia responses: implications for nanotherapeutics

**DOI:** 10.3389/fimmu.2025.1605669

**Published:** 2025-07-28

**Authors:** Yimin Wang, Fang Zhou

**Affiliations:** ^1^ Department of Hematology, the 960th Hospital of the People’s Liberation Army Joint Logistics Support Force, Jinan, China; ^2^ The First Clinical Medical School, Shandong University of Traditional Chinese Medicine, Jinan, China

**Keywords:** HIF-1α, chronic graft-versus-host disease, immunomodulation, fibrosis, graft-versus-leukemia, nanomedicine

## Abstract

Chronic graft-versus-host disease (cGVHD), a severe complication of allogeneic hematopoietic stem cell transplantation (allo-HSCT), arises from donor immune cell-mediated tissue damage, chronic inflammation, and fibrosis. Current therapies fail to adequately address fibrotic progression and heighten infection risks, underscoring the need for targeted strategies. Hypoxia-inducible factor-1α (HIF-1α), a pivotal regulator, emerges as a potential therapeutic target by orchestrating immunometabolic homeostasis, suppressing fibrosis, preserving gut microbiota balance, and retaining graft-versus-leukemia (GVL) effects. However, clinical translation necessitates overcoming challenges in tissue specificity and off-target effects. Smart nanodelivery systems hold promise for enhancing precision to enable localized HIF-1α pathway modulation. This review highlights the multidimensional roles of HIF-1α in cGVHD pathogenesis and proposes nanotherapeutic approaches to reconcile immunofibrotic imbalances, advancing a paradigm shift in cGVHD management while preserving GVL efficacy.

## 1 Introduction

Chronic graft-versus-host disease (cGVHD), a lethal complication following allogeneic hematopoietic stem cell transplantation (allo-HSCT), is characterized by a core pathological mechanism involving persistent attack of donor-derived immune cells on host tissues, which triggers chronic inflammation, immune tolerance dysregulation, and multi-organ fibrosis. Post-transplantation, donor immune cells infiltrate host tissues and induce cellular damage, releasing damage-associated molecular patterns (DAMPs) and pathogen-associated molecular patterns (PAMPs) (1, 2). These molecules activate innate immune receptors including Toll-like receptors (TLRs), NOD-like receptors, and the NLRP3 inflammasome, thereby driving pro-inflammatory cytokine cascades (3). The chronic inflammatory microenvironment facilitates antigen-presenting cells (APCs) to activate autoreactive B and T lymphocytes. Concurrently, compromised central and peripheral immune tolerance mechanisms exacerbate the targeting of donor-recipient shared antigens by these cells, recapitulating autoimmune pathological processes (4).

In cGVHD, autoreactive CD4+ T cells differentiate into T helper 1(Th1), Th2, and Th17 subsets, with Th17 cells exacerbating chronic inflammation and tissue remodeling through aberrant interleukin-17 (IL-17) secretion (5). Concurrently, follicular helper T cells (Tfh), via IL-21 signaling, orchestrate germinal center formation to drive B cell somatic hypermutation and pathogenic antibody production (6). Sustained immune dysregulation promotes macrophage-derived profibrotic factors, including transforming growth factor-β (TGF-β) and platelet-derived growth factor-A (PDGF-A), which activate fibroblasts and enhance collagen deposition, ultimately culminating in organ fibrosis (7, 8). cGVHD manifests with heterogeneous clinical presentations ranging from lichenoid skin lesions and Sjögren-like syndrome to fibrotic involvement of joints, lungs, and fasciae. Approximately 20% of patients develop debilitating sclerotic skin lesions and bronchiolitis obliterans syndrome (BOS), which are hallmarks of progressive fibrotic lesions (9).

Although glucocorticoids combined with calcineurin inhibitors remain first-line therapy, approximately 50% of patients develop steroid-refractory or relapsed disease, while prolonged immunosuppression predisposes to life-threatening infections, metabolic disorders, and osteoporosis (10–12). Current therapeutic strategies demonstrate limited efficacy against progressive fibrotic complications (e.g., BOS and dermal sclerosis), with fibrotic organ failure constituting the predominant cause of cGVHD-related mortality. These clinical challenges underscore the urgent need for mechanistic dissection of cGVHD pathogenesis and development of novel targeted interventions.

Hypoxia-inducible factor-1α (HIF-1α), a master regulator of cellular adaptation to hypoxic microenvironments, has been shown to regulate Th17 differentiation (via RORγt activation), inhibit Tfh-B cell crosstalk (by modulating CD40/CD40L signaling), and reverse macrophage profibrotic polarization (through mTORC1/TGF-β axis suppression) (13–15). Furthermore, HIF-1α synergistically maintains immune balance via metabolic reprogramming, epigenetic regulation, and gut microbiota homeostasis. Notably, HIF-1α exerts protective effects against cGVHD while preserving graft-versus-leukemia (GVL) activity, offering a unique therapeutic advantage for balancing cGVHD suppression and leukemia relapse prevention.

This review systematically elucidates the multidimensional roles of HIF-1α in modulating immune-metabolic homeostasis, suppressing fibrosis, maintaining gut microecology, and sustaining GVL efficacy, thereby providing a mechanistic foundation for targeted cGVHD interventions with preserved antileukemic effects. However, clinical translation faces challenges: tissue-specific modulation, off-target effect risks, and synergistic strategies with conventional therapies remain imperative to explore. The development of smart nanodelivery systems may enhance targeting precision. Collectively, HIF-1α-targeted therapy holds promise for overcoming the dual immunofibrotic pathological barriers in cGVHD, establishing a breakthrough therapeutic paradigm for clinical translation.

## 2 Introduction to HIF-1α

### 2.1 Structure of HIF-1α

HIF-1α, the oxygen-sensitive subunit of the heterodimeric transcription factor HIF-1, is a master regulator of cellular adaptation to hypoxia. The human HIF-1α protein consists of 826 amino acids encoded by the HIF1A gene located on chromosome 14q21-24 (16, 17). Structurally, HIF-1α belongs to the basic helix-loop-helix (bHLH)/PER-ARNT-SIM (PAS) superfamily. Its N-terminal region contains a bHLH domain essential for DNA binding and a PAS domain mediating heterodimerization with the constitutively expressed HIF-1β/ARNT subunit (18, 19). The central region contains the oxygen-dependent degradation domain (ODDD), which is characterized by two critical proline residues (Pro402 and Pro564) targeted by prolyl hydroxylases (PHDs). Hydroxylation of these residues triggers VHL-dependent ubiquitination and proteasomal degradation under normoxia. The C-terminal region harbors two transactivation domains (TAD-N and TAD-C) and an oxygen-regulated inhibitory domain (CID). TAD-N and TAD-C synergistically activate hypoxia-responsive genes through recruitment of transcriptional coactivators such as p300/CBP (20). Notably, TAD-C activity is further regulated by asparagine hydroxylation under normoxia, which prevents coactivator binding.

### 2.2 Physiological functions of HIF-1α

HIF-1α enhances cellular resilience to hypoxia by suppressing apoptosis via upregulation of B-cell lymphoma-2 (BCL-2) family proteins and inhibition of pro-apoptotic factors. Simultaneously, it sustains proliferation by activating mitogenic pathways such as IGF-2/IGF-1R signaling (21, 22). HIF-1α orchestrates inflammatory responses by polarizing macrophages toward a pro-inflammatory phenotype (M1) and enhancing neutrophil survival. It amplifies cytokine production (IL-1β, TNF-α, IL-6) and regulates T cell differentiation, linking hypoxia to chronic inflammation and autoimmune pathologies (23). HIF-1α stimulates erythropoiesis by inducing erythropoietin (EPO) in renal interstitial cells, optimizing oxygen delivery during systemic hypoxia (24). The pleiotropic roles of HIF-1α extend to cancer progression, cardiovascular remodeling, and inflammatory diseases. Its dysregulation contributes to tumor angiogenesis, metabolic adaptation, and therapy resistance, positioning it as a promising therapeutic target. Emerging strategies aim to modulate HIF-1α stability or activity using small-molecule inhibitors or gene-editing approaches (25, 26).

## 3 HIF-1α and immune regulation

### 3.1 T cells

#### 3.1.1 T cells and cGVHD

In cGVHD, the specific functions of T cells include direct cytotoxic effects, cytokine production, and regulation of other immune cells. After allo-HSCT, donor T cells may recognize recipient tissue antigens and become activated. These activated T cells can differentiate into effector T cells and memory T cells, with effector T cells capable of directly attacking the host’s tissues, leading to clinical symptoms of cGVHD. Donor CD4+ and CD8+ T cells can cause significant cGVHD symptoms, with CD8+ T cell-mediated cGVHD being thymus-dependent and preferentially damaging the recipient’s thymic medullary epithelial cells, resulting in defects in thymic negative selection (27). In cGVHD, the number and function of regulatory T cells (Tregs) may be impaired, and a reduction in Tregs is associated with the development of extensive cGVHD. For example, the imbalance in the reconstitution of CD4+ Tregs and conventional CD4+ T cells (CD4+ Tcon) after allo-HSCT promotes cGVHD progression. Additionally, Tregs can inhibit T cell function by secreting immunosuppressive factors such as IL-10 and TGF-β, as well as inducing apoptosis in effector T cells through perforin and granzyme secretion (28, 29). The interaction between T cells and B cells is also critical in cGVHD. Tfh interacting with B cells can promote B cell differentiation and antibody production. In cGVHD patients, the expression of circulating follicular helper T (cTfh) cells, extrafollicular helper T cells, and B cells is altered, and these changes correlate with cGVHD pathogenesis. For instance, cTfh cell levels are significantly reduced in cGVHD patients compared to controls, whereas extrafollicular helper T cells are markedly elevated (30, 31). Furthermore, various cytokines contribute to cGVHD by regulating T cell function and differentiation. TGF-β1 promotes Foxp3 expression in T cells and induces Th cell conversion and expansion into Tregs via the SMAD signaling pathway (32). Additionally, TGF-β1 enhances CTLA-4 expression in naïve T cells, thereby promoting Foxp3 production in Tregs (33). IL-2 therapy can restore CD4+ T cell subset homeostasis in cGVHD patients and promote immune tolerance reconstitution (34). Given the pivotal role of T cells in cGVHD, they represent potential therapeutic targets.

#### 3.1.2 HIF-1α and T cells

**Figure 1:**
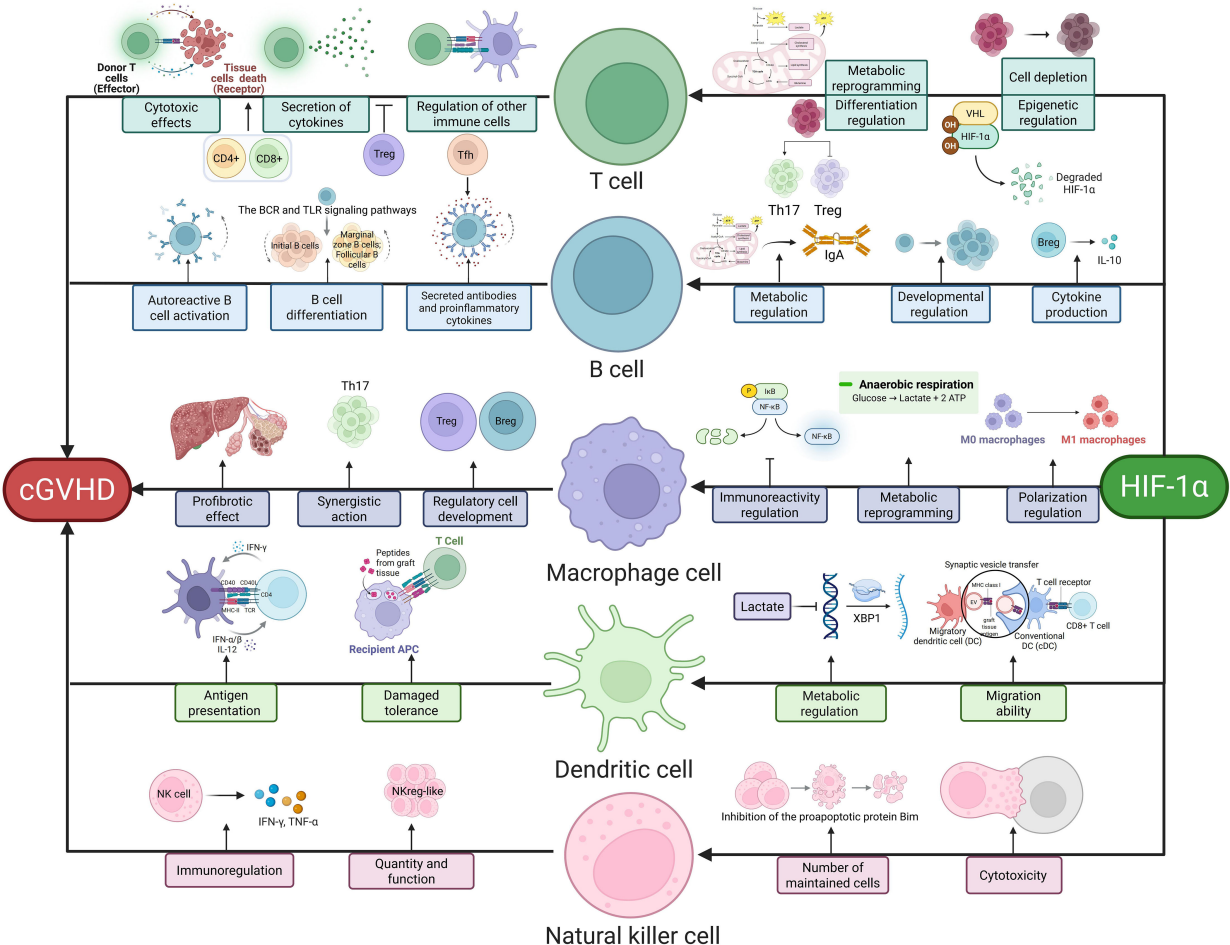
HIF-1α-Mediated Immune Regulation in cGVHD. This schematic delineates key immune pathways modulated by HIF-1α: T cells drive pathology via cytotoxic effects (CD8^+^-mediated thymic epithelial damage), cytokine secretion (Th17/Treg imbalance), and immune regulation (impaired Treg suppression via IL-10/TGF-β). B cells contribute through autoreactive activation (BCR/TLR signaling), antibody/proinflammatory cytokine secretion (e.g., anti-PDGFR antibodies), and regulatory dysfunction (Breg-derived IL-10 deficiency). Macrophages promote fibrosis via profibrotic polarization (TGF-β secretion), metabolic reprogramming, and amplified inflammation (M1/M2 imbalance). Dendritic cells facilitate antigen presentation (host antigen-driven T cell activation), impaired tolerance (reduced Treg induction), and migratory defects (HIF-1α/CCR7 axis). NK cells regulate disease through cytotoxic activity (granzyme/perforin), quantitative/functional alterations, and subset-specific modulation (NKreg-like suppression vs. CD74^+^-mediated inflammation). Central to these pathways, HIF-1α orchestrates metabolic reprogramming (glycolysis induction via PKM2/LDHA), epigenetic regulation (VHL/PHD axis), and cytokine signaling, thereby integrating immune dysregulation in cGVHD pathogenesis.

HIF-1α plays a critical role in T cell differentiation and function. It is central to T cell metabolic reprogramming. Under hypoxia, HIF-1α promotes a metabolic shift from oxidative phosphorylation to glycolysis in T cells, a process essential for their activation and effector functions. For example, HIF-1α deficiency prevents metabolic reprogramming in hypoxic T cells, thereby inhibiting IFN-γ induction (35). In chronic infections and tumor microenvironments, T cells may undergo exhaustion, characterized by functional decline and upregulated inhibitory receptors. HIF-1α regulates this process. Studies show that mitochondrial dysfunction reduces α-ketoglutarate levels, thereby inhibiting PHD activity and stabilizing HIF-1α in progenitor exhausted T cells (Tpex), driving their transcriptional and metabolic reprogramming into terminally exhausted T cells (36). Moreover, HIF-1α deficiency impairs glycolytic flux, leading to reduced ATP production and compromised activation-induced cell death (AICD) in hypoxic T cells (35). HIF-1α also regulates T cell differentiation into subsets such as Th1, Th2, Th17, and Tregs. In Th17 differentiation, HIF-1α acts via retinoic acid-related orphan receptor-γt (RORγt) to drive this process (37). Specific HIF-1α knockout in Tregs enhances their suppressive capacity under hypoxia, potentially by promoting oxidative phosphorylation and upregulating inhibitory receptors such as CTLA-4 (38). HIF-1α further participates in T cell epigenetic regulation. Under normoxia, HIF-1α undergoes hydroxylation by prolyl hydroxylases (PHD1-3), triggering polyubiquitination and proteasomal degradation via the von Hippel-Lindau protein (pVHL) complex (39). HIF-1α also regulates T cell oxidative stress responses. Hypoxia stabilizes HIF-1α, which modulates glycolysis-related gene expression to influence ATP and ROS production, thereby promoting T cell metabolic adaptation and survival (40). Elucidating its specific mechanisms may aid in developing novel immunotherapies for cGVHD (**Figure 1**).

### 3.2 B cells

#### 3.2.1 B cells and cGVHD

The roles and mechanisms of B cells in cGVHD are multifaceted, involving activation, differentiation, and specific pathological effects. In cGVHD, B cell activation primarily occurs through B cell receptor (BCR) and Toll-like receptor (TLR) signaling. Key molecules in the BCR pathway, such as B cell linker protein (BLNK) and spleen tyrosine kinase (SYK), are upregulated in cGVHD patients. This activation promotes autoreactive B cell survival and maturation via NF-κB and ERK signaling (41–43). B cell differentiation is also critical in cGVHD. Studies reveal reduced proportions and counts of naïve and immature B cells in cGVHD patients, alongside increased marginal zone and follicular B cells, which correlate with disease progression (30). Regulatory B cells (Bregs) modulate immune responses in cGVHD by maintaining immune balance, inducing transplant tolerance, and exerting anti-cGVHD effects. Bregs secrete cytokines like IL-10 to inhibit T cell and macrophage activation, thereby reducing inflammation and immune attacks (44, 45). Autoreactive B cell survival depends on B cell-activating factor (BAFF) levels: low BAFF promotes their clearance, while high BAFF enhances survival by activating AKT and ERK signaling (46, 47). In cGVHD, B cells promote autoimmunity through host-reactive antibody secretion, pro-inflammatory cytokine production, and self-antigen presentation to T cells. Anti-PDGFR stimulatory antibodies found in cGVHD patients activate ROS production and type I collagen gene expression (48). In females, post-transplant detection of allo-HY antibodies predicts human cGVHD (49). Additionally, B cells from active cGVHD patients exhibit impaired IL-10 production (50). The complex roles of B cells in cGVHD highlight the need for mechanistic studies to develop novel therapies.

#### 3.2.2 HIF-1α and B cells

HIF-1α is critical for B cell differentiation, function, and immune regulation. Studies show that HIF-1α regulates IgA-producing B cell differentiation via metabolic reprogramming. HIF-1α-deficient B cells exhibit reduced glycolysis and impaired IgA production, whereas PHD inhibitor roxadustat stabilizes HIF-1α to enhance IgA class switching and mitigate inflammation (51). HIF-1α activity is dynamically regulated during B cell development. Bone marrow pro-B and pre-B cells exhibit high HIF activity, which declines at the immature B cell stage. Genetic HIF-1α activation in B cells reduces clonal diversity, impairs BCR editing, and arrests immature B cell development, decreasing peripheral B cell numbers. HIF-1α activation also reduces surface BCR, CD19, and BAFF-R expression while increasing pro-apoptotic Bim levels (52). B cell-specific HIF-1α knockout impairs IL-10 production, and HIF-1α cooperates with phosphorylated STAT3 to regulate IL-10 expression. HIF-1α-dependent glycolysis promotes CD1dhiCD5+ B cell expansion. Compared to wild-type mice, HIF-1α-deficient B cell mice exhibit exacerbated collagen-induced arthritis (CIA) and experimental autoimmune encephalomyelitis (EAE), which can be rescued by IL-10-expressing CD1dhiCD5+ B cell transplantation (53). In rheumatoid arthritis (RA), the CD27 IgD naïve B cell subset produces IL-6, and both HIF-1α and IL-6 are co-expressed in RA patient B cells. HIF-1α directly binds the IL6 promoter to enhance its transcription (54). By regulating metabolism, cytokine production, and immune cell development, HIF-1α maintains immune balance. Further research into its mechanisms may advance therapeutic strategies for related diseases (**Figure 1**).

### 3.3 Macrophages

#### 3.3.1 Macrophages and cGVHD

Macrophages play a key role in the pathogenesis of cGVHD, contributing to its occurrence, progression, and pathological mechanisms. They induce fibroblast differentiation into myofibroblasts by secreting pro-fibrotic cytokines such as TGF-β, which promote collagen synthesis and deposition, ultimately leading to tissue fibrosis (55). Additionally, macrophages interact with T cells through cytokine secretion and co-stimulatory molecule expression, enhancing T cell activation and proliferation. This interaction particularly drives Th17 cell formation, thereby creating a positive feedback loop that exacerbates cGVHD pathology (56). Macrophages also influence the development and function of Tregs and Bregs; their dysfunction may reduce the number or impair the function of these regulatory subsets, disrupting immune tolerance and triggering uncontrolled immune responses in cGVHD (47). During the transition from acute graft-versus-host disease (aGVHD) to cGVHD, macrophages release inflammatory mediators and reactive oxygen species (ROS), directly damaging host tissues and organs and establishing the foundation for cGVHD onset (57). In chronic cGVHD, M2 macrophages promote tissue fibrosis via TGF-β secretion, resulting in organ dysfunction and amplified inflammatory responses (58). Given these multifaceted roles, macrophage-targeted therapies—such as anti-CSF-1R antibodies and the anti-fibrotic drug pirfenidone—hold significant clinical potential, effectively alleviating pathological damage and improving patient prognosis (59). Further research into macrophage-mediated mechanisms in cGVHD is critical for developing more effective treatments.

#### 3.3.2 HIF-1α and macrophages

HIF-1α is central to macrophage biology, regulating immune responses, migration, polarization, and metabolism. It modulates macrophage immune activity by both inhibiting NF-κB transcriptional activity to prevent excessive inflammation and promoting NF-κB-mediated pro-inflammatory cytokine expression during LPS stimulation to bolster host defense (60, 61). HIF-1α enhances macrophage phagocytosis by suppressing apoptosis and PHD activity, thereby stabilizing its own protein levels. It also improves macrophage antibacterial capacity by regulating antimicrobial peptide release and nitric oxide (NO) production (62). In migration, HIF-1α fuels macrophages via aerobic glycolysis induction. Under severe hypoxia, HIF-1α upregulates pyruvate dehydrogenase kinase 1 (PDK1) to block pyruvate entry into the tricarboxylic acid cycle, thereby regulating glucose oxidation and enhancing migratory potential (63, 64). HIF-1α further drives macrophage polarization toward the pro-inflammatory M1 phenotype by targeting glucose metabolism. Activation of the HIF-1α/pyruvate kinase M2 (PKM2) axis promotes M1 polarization and inflammatory factor release (65, 66). In metabolic reprogramming, HIF-1α is a key regulator of macrophage glycolysis, binding to hypoxia response elements (HREs) in glycolytic enzyme gene promoters to upregulate their expression and drive aerobic glycolysis (67). Additionally, HIF-1α-induced upregulation of glycolytic enzymes and tricarboxylic acid cycle intermediates stabilizes HIF-1α itself, forming a positive feedback loop. LPS-induced disruption of the tricarboxylic acid cycle leads to metabolite accumulation (e.g., succinate, fumarate), which directly inhibits PHD activity and stabilizes HIF-1α (68, 69) (**Figure 1**).

### 3.4 Dendritic cells

#### 3.4.1 Dendritic cells and cGVHD

Dendritic cells (DCs) exhibit a dual immunoregulatory role in cGVHD, contributing to both disease progression and immune homeostasis. As professional APC, DCs initiate pathogenic immune responses by capturing and presenting host-derived antigens to donor T cells, thereby activating alloreactive immune pathways (70). Following transplantation, donor-derived DCs rapidly infiltrate recipient tissues, where they secrete proinflammatory cytokines and express co-stimulatory molecules to drive donor T cell activation and proliferation. This process preferentially promotes Th1/Th17 cell differentiation, amplifying inflammatory cascades that exacerbate tissue damage (71). Paradoxically, DCs possess intrinsic tolerogenic potential under physiological conditions, capable of maintaining immune equilibrium through Tregs induction and functional enhancement (72). However, cGVHD pathogenesis involves critical dysfunction of these tolerogenic mechanisms. The impaired ability of DCs to generate and sustain Tregs populations results in progressive loss of immune tolerance, a hallmark of chronic disease progression (73). Post-allo-HSCT complications further compound this imbalance: conditioning regimens and acute GVHD (aGVHD)-mediated thymic damage disrupt central tolerance mechanisms, while peripheral DC dysfunction permits the escape of autoreactive T cell clones (57). Donor DCs in peripheral tissues perpetuate this cycle through indirect antigen presentation, continuously activating pathogenic T cell populations and driving cGVHD progression (74). This dual functionality positions DCs as central orchestrators of the cGVHD paradox—simultaneous hyperactivation of effector responses and failure of tolerance mechanisms. Emerging therapeutic strategies aim to recalibrate DC function through two complementary approaches: pharmacological modulation of DC activation thresholds and ex vivo generation of tolerogenic DC subsets. These interventions seek to restore the delicate balance between immunogenic and tolerogenic DC activities, offering novel pathways for clinical management of cGVHD.

#### 3.4.2 HIF-1α and dendritic cells

HIF-1α serves as a master regulator of dendritic cell (DC) biology, coordinating metabolic adaptation, functional polarization, and migratory behavior. Lactate-mediated stabilization of HIF-1α induces NDUFA4L2 expression, which suppresses mitochondrial reactive oxygen species (ROS) production and subsequently inhibits the XBP1-dependent transcriptional program that drives pathogenic autoimmune T cell responses (75). Hypoxic conditioning studies reveal that HIF-1α-deficient DCs exhibit impaired IL-22 secretion and reduced CCR7 chemokine receptor expression, resulting in defective migratory capacity. These findings establish HIF-1α as an essential mediator of DC differentiation and tissue homing under low oxygen conditions (76). The long non-coding RNA lnc-Dpf3 directly interacts with HIF-1α to repress transcription of the glycolytic enzyme gene Ldha, effectively dampening DC glycolytic flux and migration capabilities (77). Magnesium ion signaling through the TRPM7 channel activates MAPK pathways in DCs, inducing HIF-1α expression that enhances TGF-β production while suppressing effector T cell function (78). Pathogen interaction studies demonstrate that Mycobacterium tuberculosis-infected DCs upregulate HIF-1α-driven aerobic glycolysis via TLR2-dependent mechanisms, a metabolic reprogramming event critical for DC migratory responses during infection (79) (**Figure 1**).

### 3.5 Natural killer cells

#### 3.5.1 Natural killer cells and cGVHD

The role of natural killer (NK) cells in cGVHD remains incompletely defined, though emerging evidence suggests their significant involvement in disease pathogenesis. Clinical studies demonstrate a clinically relevant association between NK cell quantity/functionality and cGVHD outcomes. Notably, patients with extensive cGVHD exhibit reduced peripheral blood NK cell counts compared to those with limited disease. Furthermore, histological analysis reveals diminished NK cell infiltration in skin lesions of cGVHD patients who underwent HLA-matched sibling allo-HSCT relative to acute GVHD cases. This observation suggests that decreased circulating NK cell numbers may reflect enhanced tissue-specific recruitment and localization to cGVHD-affected sites (80). The therapeutic potential of NK cell modulation is highlighted by clinical outcomes in umbilical cord blood (UCB) transplantation. Pediatric UCB transplant recipients demonstrate both significantly higher NK cell reconstitution and reduced cGVHD incidence compared to other donor sources. Early detection of functionally competent NK cells post-transplantation has been identified as a critical factor contributing to this protective effect against cGVHD development (81). Mechanistically, NK cells appear to participate in complex immune regulatory networks during cGVHD pathogenesis. While they may collaborate with inhibitory CD8+ T cells to suppress autoantibody-producing B cells, activation through the MICA-NKG2D axis appears detrimental. Elevated plasma levels of soluble MICA correlate with increased cGVHD susceptibility, suggesting therapeutic potential in targeting IFN-γ production via this pathway (82). Emerging research identifies specific NK cell subsets with distinct functional roles in cGVHD modulation. A CD56bright perforin-regulatory NK cell population (NKreg-like) demonstrates potent immunosuppressive capacity, with increased numbers correlating with cGVHD suppression. This subset represents a promising candidate for cell-based therapeutic strategies (83). Conversely, CD74+ NK cells exhibit pro-inflammatory characteristics through high mitochondrial potential and cytokine secretion (IFN-γ/TNF-α), driving pulmonary cGVHD pathogenesis via CXCL10-mediated recruitment of macrophages and CD4+ T cells. Preclinical models demonstrate that targeted depletion of CD74+ NK cells using anti-CD74 antibodies effectively mitigates lung injury while preserving CD74- NK cell populations to maintain GVL effects (84). These findings collectively underscore the dual regulatory potential of NK cells in cGVHD pathophysiology. While certain subsets contribute to tissue damage through inflammatory mechanisms, others demonstrate protective immunosuppressive properties. This dichotomy emphasizes the necessity for precise subset-specific modulation strategies to optimize NK cell-mediated benefits in cGVHD management, balancing therapeutic efficacy with preservation of anti-neoplastic activity.

#### 3.5.2 HIF-1α and natural killer cells

HIF-1α has been demonstrated to play a multifaceted regulatory role in natural killer (NK) cell metabolism, anti-tumor immunity, and infection response under hypoxic conditions. Emerging evidence reveals a complex interplay between HIF-1α and cytokine signaling pathways in the tumor microenvironment. Multiple studies indicate that HIF-1α suppresses NK cell-mediated tumor surveillance by inhibiting the IL-18-dependent NF-κB signaling axis, thereby attenuating anti-tumor responses. Notably, HIF-1α-deficient NK cells exhibit enhanced tumoricidal activity characterized by elevated IFN-γ production, increased granzyme B expression, and amplified degranulation capacity, suggesting therapeutic potential through HIF-1α inhibition (85). Contrasting findings highlight the context-dependent nature of HIF-1α function in NK cell biology. Paradoxically, other investigations demonstrate that HIF-1α deficiency impairs NK cell cytotoxicity by reducing infiltration of cells expressing soluble VEGFR-1, an anti-angiogenic factor critical for suppressing tumor-associated neovascularization. This deficiency leads to dysfunctional angiogenesis despite attenuated tumor progression (86). These opposing outcomes underscore the dual regulatory roles of HIF-1α in modulating both direct cytotoxic mechanisms and microenvironmental interactions. The cytokine milieu further modulates HIF-1α activity through distinct signaling pathways. IL-15 maintains NK cell homeostasis and effector functions through STAT3-mediated stabilization of HIF-1α, while IL-2 promotes HIF-1α expression via PI3K/mTOR signaling activation. These molecular mechanisms collectively preserve NK cell-mediated defenses against malignant transformation and pathogenic challenges (87, 88). Such cytokine-HIF-1α crosstalk appears essential for maintaining metabolic fitness and functional competence under hypoxic stress. Recent mechanistic insights reveal an essential metabolic regulatory role of HIF-1α in NK cell survival during viral infections. HIF-1α-dependent metabolic reprogramming suppresses expression of the pro-apoptotic protein Bim, thereby maintaining adequate NK cell populations required for optimal antiviral responses. This survival mechanism highlights the evolutionary conservation of HIF-1α-mediated metabolic adaptation in preserving lymphoid cell homeostasis during infection (89). Collectively, these findings position HIF-1α as a critical molecular nexus integrating environmental signals, metabolic demands, and functional outputs in NK cell biology (**Figure 1**).

## 4 HIF-1α and gut microbiota

### 4.1 Gut microbiota and cGVHD

Gut microbiota dysbiosis is closely linked to the pathogenesis of cGVHD. Significant alterations in gut microbiota composition are observed in cGVHD patients, notably a reduction in beneficial bacteria and an expansion of potential pathogens. The decline of butyrate-producing bacteria (e.g., Lachnoclostridium, Clostridium, and Faecalibacterium) may impair intestinal barrier integrity, thereby exacerbating cGVHD progression (90). Gut microbiota-derived metabolites also play critical roles in cGVHD. Short-chain fatty acids (SCFAs) enhance Tregs differentiation and function by inhibiting histone deacetylase (HDAC) activity, thereby mitigating cGVHD severity (91, 92). Tryptophan metabolites such as indole and its derivatives regulate Treg/Th17 balance via aryl hydrocarbon receptor (AhR) activation, suppressing inflammation and promoting intestinal epithelial repair (93, 94). Bile acids inhibit pro-inflammatory cytokines (TNF-α, IL-12) in DCs and monocytes while stimulating IL-10 production in macrophages, thus dampening immune cell pro-inflammatory activity. Bile acids also modulate Treg/Th17 equilibrium (95, 96). Secondary bile acids like 3-oxoLCA (3-oxo lithocholic acid) and isoalloLCA directly bind the transcription factor RORγt to inhibit Th17 differentiation, whereas isoalloLCA enhances Tregs differentiation by promoting oxidative phosphorylation and CNS3 H3K27 acetylation (97). Interventions targeting gut microbiota, such as fecal microbiota transplantation (FMT), have demonstrated therapeutic potential in cGVHD (98, 99).

### 4.2 HIF-1α and gut microbiota

**Figure 2:**
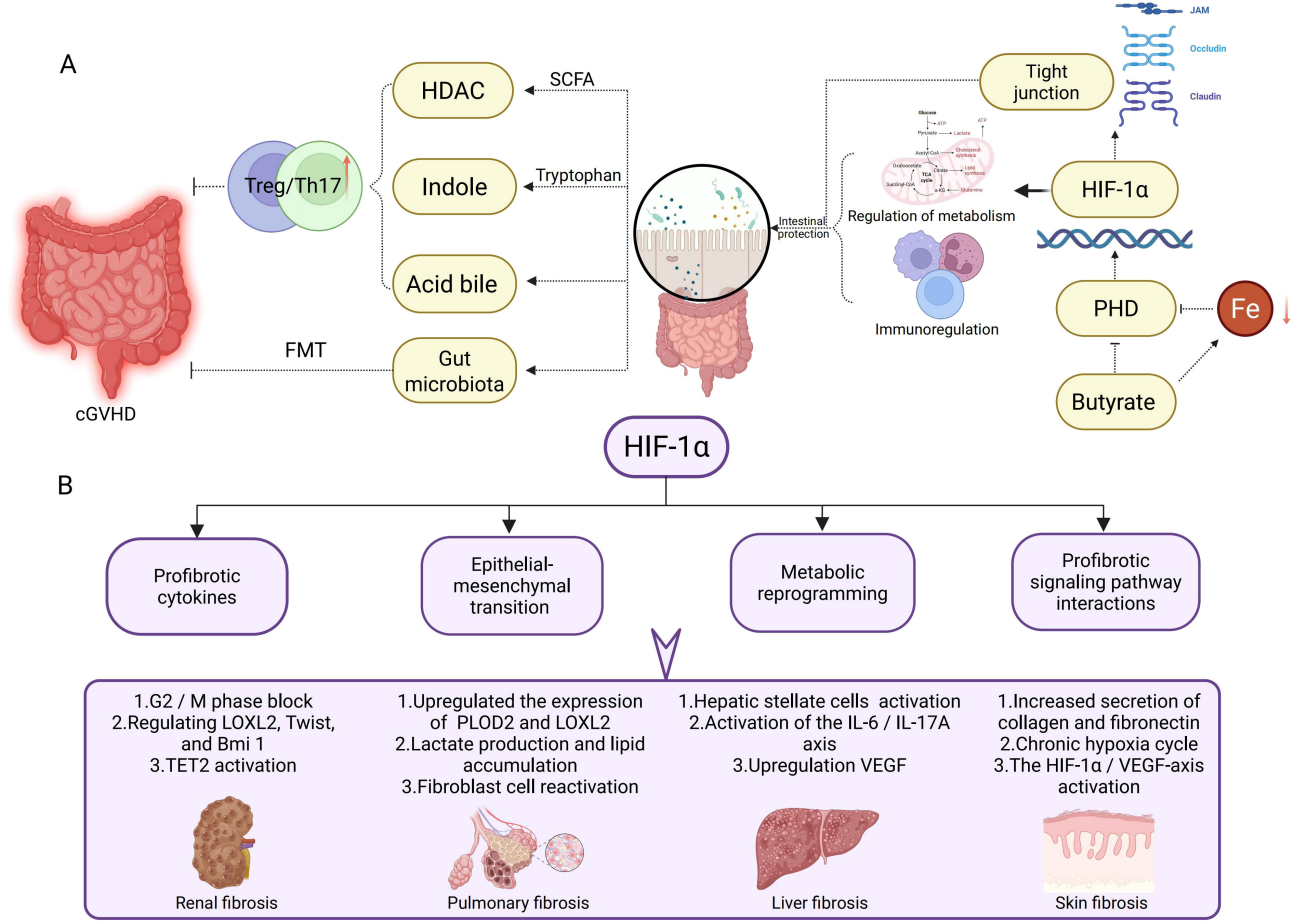
HIF-1α in Gut Microbiota Regulation and Tissue Fibrosis Pathogenesis. **(A)** This section illustrates the potential mechanisms of the gut microbiota in cGVHD via HIF-1α. Short-chain fatty acids, indoles, and bile acids modulate the Treg/Th17 cell balance, influencing intestinal barrier integrity and immune regulation. HIF-1α enhances tight junction protein expression to promote intestinal barrier protection, while concurrently regulating metabolic and immune responses to maintain gut homeostasis. **(B)** This section delineates the role of HIF-1α in distinct fibrotic processes. In renal fibrosis, HIF-1α induces pathogenesis via G2/M phase arrest, modulation of LOXL2, Twist, and Bmi1 expression, and TET2 activation. In pulmonary fibrosis, it mediates progression through upregulation of PLOD2 and LOXL2, lactate production, lipid accumulation, and fibroblast reactivation. Liver fibrosis is promoted by HIF-1α via hepatic stellate cell activation, the IL-6/IL-17A axis, and VEGF upregulation. In skin fibrosis, HIF-1α drives pathogenesis by enhancing collagen and fibronectin secretion, perpetuating chronic hypoxia cycles, and activating the HIF-1α/VEGF axis.

The gut microbiota modulates HIF-1α expression and stability through metabolic regulation of intestinal epithelial cells (IECs), particularly by influencing cellular oxygen consumption patterns. HIF-1α enhances intestinal barrier integrity by upregulating tight junction protein expression in IECs, creating a bidirectional regulatory axis between host physiology and microbial communities (100, 101). This transcription factor further coordinates IEC metabolic programming and immune responses, indirectly shaping gut microbiota composition and functionality. Microbial-derived SCFAs, particularly butyrate, stabilize HIF-1α through dual mechanisms: direct inhibition of PHD enzyme activity and iron chelation-mediated PHD functional impairment, both contributing to reduced HIF-1α degradation (100, 102). Post-allo-HSCT complications reveal HIF-1α’s critical protective role. Donor T cell-induced intestinal damage impairs IEC oxidative phosphorylation, paradoxically increasing luminal oxygen levels and destabilizing HIF-1α. IEC-specific HIF-1α knockout murine models demonstrate exacerbated acute graft-versus-host disease (aGVHD) severity, highlighting the protein’s essential functions in barrier maintenance and immune regulation (103). Mechanistically, HIF-1α mitigates aGVHD through three interconnected pathways: promoting IEC regeneration, modulating immune cell infiltration/activation, and maintaining beneficial microbial communities. These findings position HIF-1α as a central regulator of intestinal homeostasis under transplant-related stress conditions (**Figure 2A**).

## 5 HIF-1α and tissue fibrosis

Fibrosis, characterized by excessive extracellular matrix (ECM) deposition, is a hallmark of cGVHD following allo-HSCT. While preclinical models of organ fibrosis (renal, pulmonary, liver, skin) provide mechanistic insights, their relevance to allo-HSCT lies in cross-pathway similarities with cGVHD-mediated tissue damage.

### 5.1 Renal fibrosis

In allo-HSCT, renal fibrosis associated with cGVHD or calcineurin inhibitor nephrotoxicity mirrors HIF-1α-driven mechanisms observed in idiopathic models. HIF-1α-induced G2/M arrest in tubular epithelial cells (via p53 upregulation) and EMT (mediated by LOXL2/Twist/Bmi1) promote TGF-β–driven ECM deposition (type I collagen, fibronectin) (104–107). Notably, TET2 activation—implicated in both fibrosis and immune regulation—links HIF-1α to epigenetic reprogramming in fibrotic kidneys (108). These mechanisms underscore HIF-1α as a potential target to mitigate cGVHD-induced renal injury (**Figure 2B**).

### 5.2 Pulmonary fibrosis

Activation of the HIF pathway induces structural and functional collagen dysregulation, a key contributor to mechanical dysfunction in idiopathic pulmonary fibrosis (IPF) tissues. Pulmonary complications post-allo-HSCT share pathogenic features with idiopathic pulmonary fibrosis (IPF). HIF upregulates enzymes such as PLOD2 and LOXL2 to alter collagen crosslinking, fiber nanostructure, and tissue stiffness (109). CCT6A, highly expressed in type II alveolar epithelial cells (AEC2) of fibrotic lungs, correlates with disease severity. It suppresses HIF-1α-mediated lactate production by promoting VHL-dependent ubiquitination and degradation of HIF-1α, thereby inhibiting lipid accumulation and alleviating bleomycin-induced pulmonary fibrosis in mice (110). Drp1 inhibition prevents mitochondrial fission in fibroblasts and regulates ROS/HIF-1α-dependent lipid metabolic reprogramming, suppressing fibroblast activation and mitigating fibrosis progression (111) (**Figure 2B**).

### 5.3 Liver fibrosis

HIF-1α also plays a critical role in liver fibrosis. Studies demonstrate that liver fibrosis and end-stage cirrhosis are frequently associated with hepatic hypoxia. As a key transcription factor regulating cellular hypoxia responses, HIF-1α is critically involved in hepatic stellate cell (HSC) activation and fibrosis progression. During liver fibrosis, HSCs respond to hypoxic stimuli, upregulating HIF-1α expression. HIF-1α promotes IL-6 production by directly binding to the hypoxia-response element (HRE) in the IL6 promoter of HSCs, which stimulates Th17 cells to secrete IL-17A. This interaction establishes the HIF-1α/IL-6/IL-17A axis, driving fibrotic development (112). HIF-1α further regulates elevated VEGF levels in activated HSCs. Concurrently, miR-21 overexpression in hepatocytes stabilizes HIF-1α via pVHL, amplifying the HIF-1α/VEGF signaling pathway and accelerating fibrosis (113). Additionally, HIF-1α interacts with key pathways—including PI3K/AKT/mTOR, MAPK/ERK, and NF-κB—all of which contribute to the onset and progression of liver fibrosis (61, 114–116) (**Figure 2B**).

### 5.4 Skin fibrosis

Systemic sclerosis (SSc), characterized by skin/visceral fibrosis and microvascular damage, shares pathogenic parallels with sclerodermatous cGVHD. HIF-1α hyperactivation in SSc perpetuates a cycle of chronic hypoxia, promotes excessive ECM production (collagen, fibronectin) by fibroblasts, and drives endothelial-to-mesenchymal transition via the HIF-1α/VEGF axis (117–119). This knowledge provides mechanistic insight into the development of sclerodermatous cGVHD, where similar hypoxic microenvironments and profibrotic signaling likely contribute to skin and internal organ fibrosis (**Figure 2B**).

## 6 HIF-1α and antileukemic effects

HIF-1α is highly expressed across multiple malignancies and correlates with malignant progression and poor patient prognosis. In hematologic cancers, HIF-1α plays a critical role in leukemogenesis, disease progression, and therapeutic resistance. Experimental evidence confirms that bone marrow (BM) oxygen levels are exceptionally low (~0.6%), creating a hypoxic microenvironment that protects leukemia stem cells and sustains their self-renewal capacity (120, 121).

### 6.1 HIF-1α and acute lymphoblastic leukemia

In acute lymphoblastic leukemia (ALL), HIF-1α functions as an oncogenic driver. Its targeted inhibition suppresses tumor proliferation, induces apoptosis, and enhances chemosensitivity. Mechanistically, deferoxamine (DFO)-mediated HIF-1α inhibition blocks tumor growth and triggers apoptosis by inactivating the ROS/HIF-1α signaling axis (122). Chemical inhibition of HIF-1α downregulates Yin-Yang 1 (YY1) expression, thereby potentiating ALL cell responsiveness to chemotherapeutic agents (123). HIF-1α knockdown in human T-ALL cells and murine models restores hypoxia-compromised mTOR activity, significantly improving chemotherapeutic sensitivity (124). Notably, Notch1 signaling is indispensable for HIF-1α-driven proliferation, invasion, and chemoresistance in T-ALL. Hypoxic HIF-1α silencing suppresses Notch1 activation, further validating HIF-1α as a promising therapeutic target for T-ALL (125). These findings collectively establish HIF-1α as a key molecular target, with therapeutic interventions against HIF-1α showing potential to optimize clinical outcomes in ALL patients.

### 6.2 HIF-1α and chronic lymphocytic leukemia

In chronic lymphocytic leukemia (CLL), HIF-1α serves as a pivotal oncogene that drives CLL cell survival and proliferation through multiple molecular mechanisms. Stromal cells upregulate HIF-1α expression in CLL cells via the CXCL12/CXCR4 signaling axis, particularly in TP53-deficient cells where HIF-1α accumulation confers apoptosis resistance and enhances chemoresistance (126, 127). Pharmacological HIF-1α inhibitors (e.g., BAY87-2243, EZN-2208) potentiate fludarabine-induced apoptosis and improve therapeutic response (128, 129). HIF-1α further orchestrates CLL cell interactions with tumor microenvironments by regulating chemotaxis and bone marrow/spleen niche adhesion (130). Under hypoxic conditions, HIF-1α overexpression amplifies adenosine biosynthesis and signaling through A2A receptors, establishing an immunosuppressive microenvironment that promotes tumor progression and drug resistance. Importantly, A2A receptor blockade reverses these effects and restores leukemic cell sensitivity to therapeutic agents (131). Mechanistically, HIF-1α inhibitors disrupt redox homeostasis by modulating oxidative stress pathways, effectively suppressing CLL cell proliferation (132). These findings collectively establish HIF-1α pathway targeting as a viable strategy to subvert CLL cell survival mechanisms, offering significant potential for improving clinical outcomes in CLL patients.

### 6.3 HIF-1α and acute myeloid leukemia

HIF-1α is an important player in the pathogenesis and progression of AML, functioning as a crucial regulator of cellular responses to hypoxia in the bone marrow microenvironment. Frequently overexpressed in AML subsets with adverse genetic abnormalities like t (8, 21) rearrangement, NPM1 mutations, and IDH1/2 or TP53 alterations, HIF-1α overexpression is strongly linked to poor clinical outcomes, including reduced relapse-free survival and resistance to chemotherapy agents such as cytarabine (133–135). The hypoxic bone marrow microenvironment stabilizes HIF-1α, which protects leukemic stem cells (LSCs) by facilitating immune evasion and chemoresistance (136). HIF-1α drives AML pathogenesis through multiple interconnected mechanisms. It promotes leukemic cell growth, proliferation, migration, and invasion. Overexpression of HIF-1α confers doxorubicin resistance in AML cells and synergizes with leukemia-derived macrophage migration inhibitory factor (MIF) to enhance cellular proliferation and survival. Targeting the HIF-1α/YAP axis enhances doxorubicin chemosensitivity by disrupting this regulatory circuit (137, 138). In acute promyelocytic leukemia (APL), HIF-1α collaborates with the PML-RARα fusion protein to maintain leukemia stem cell self-renewal and promote tumor neovascularization and migration (139). HIF-1α enhances glycolytic metabolism, promotes genomic hypermethylation and histone modifications, regulates autophagy, and inhibits apoptosis. It also mediates chemoresistance by causing cell cycle arrest at the G0/G1 phase and forming regulatory loops with proteins like YAP (137, 140). Additionally, factors such as PARP14 and hypoxia-induced CXCL2 signaling can modulate HIF-1α activity to support AML progression (141).

Targeting HIF-1α represents a promising therapeutic strategy for AML. Inhibitors like PX-478 and echinomycin have shown potential in preclinical models by sensitizing AML cells to chemotherapy (142, 143). Hypoxia-activated prodrugs such as TH-302 have demonstrated some efficacy in clinical trials, but challenges remain in terms of toxicity and isoform-specific targeting (144). Combinatorial approaches, including HIF-1α inhibition with hypomethylating agents or BCL-2 inhibitors, may overcome resistance. Besides, elevated HIF-1α expression correlates with poor prognosis and cytarabine resistance in NPM1-mutated/FLT3-ITD-negative AML subtypes, establishing it as a predictive biomarker (133). Therapeutic HIF-1α suppression emerges as a promising strategy to restore chemosensitivity and improve clinical outcomes, positioning HIF-1α as a high-value molecular target for precision AML therapy.

### 6.4 Chronic myeloid leukemia

HIF-1α functions as a critical transcriptional regulator in chronic myeloid leukemia (CML), driving leukemic cell survival and proliferation through upregulation of BCR-ABL and Met oncogenic pathways (145). Clinically, elevated HIF-1α expression correlates with tyrosine kinase inhibitor (TKI) resistance in CML, significantly compromising therapeutic efficacy (146). Mechanistic studies reveal that HIF-1α pathway inhibition enhances TKI sensitivity and induces apoptosis through multiple modalities: hydroxyurea suppresses HIF-1α-mediated glycolytic reprogramming to overcome drug resistance, while 2-methoxyestradiol directly targets HIF-1α to trigger apoptotic cascades. The triple kinase inhibitor Tri-CAP further disrupts pro-survival signaling networks in CML cells (146–148). Importantly, HIF-1α overexpression serves as a biomarker for advanced disease progression in CML patients (149). These findings collectively establish HIF-1α as a high-value therapeutic target, offering novel strategies to optimize clinical outcomes in CML management (**Table 1**).

**Table 1:** HIF-1α and antileukemic effects.

Leukemia type​	Pathogenic role of HIF-1α​	Molecular mechanisms​	Targeted interventions​	Clinical implications​
ALL (122–125)	Oncogenic driver promoting proliferation, invasion, and chemoresistance	1.ROS/HIF-1α signaling activation 2.YY1 expression upregulation 3.Hypoxia-compromised mTOR activity 4.Notch1-dependent chemoresistance	1.Deferoxamine 2.HIF-1α/Notch1 axis blockade	Therapeutic inhibition enhances chemosensitivity and apoptosis
CLL (126–132)	Pivotal oncogene supporting survival, proliferation, and microenvironment interactions	1.CXCL12/CXCR4 axis-mediated accumulation (TP53-deficient) 2.Adenosine/A2A receptor immunosuppression 3.Redox homeostasis disruption 4.Niche adhesion regulation	1.BAY87-2243, EZN-2208 2.A2AR antagonists 3.HIF-1α inhibition + fludarabine	Reverses apoptosis resistance and improves drug response
AML (133–144)	Key regulator of LSC maintenance, metabolism, and chemoresistance in hypoxic BM	1.Immune evasion facilitation 2. HIF-1α/YAP axis-mediated doxorubicin resistance 3. Glycolysis enhancement/hypermethylation 4. Autophagy regulation/PML-RARα collaboration	1.PX-478, echinomycin 2.TH-302 (hypoxia-activated prodrug) 3.HIF-1α + HMA/BCL-2 inhibitor combos	Predicts poor prognosis; targeting restores chemosensitivity (esp. in NPM1mut/FLT3-ITD- AML)
CML (145–149)	Critical transcriptional regulator driving survival via BCR-ABL/Met pathways	1.Glycolytic reprogramming 2.Pro-survival signaling activation 3. TKI resistance mediation	1.Hydroxyurea (glycolysis suppression) 2.2-Methoxyestradiol (direct HIF-1α targeting) 3.Tri-CAP kinase inhibitor	Biomarker for advanced disease/TKI resistance; targeting enhances apoptosis and TKI efficacy

A2AR, Adenosine A2A receptor; ALL, Acute Lymphoblastic Leukemia; AML, Acute Myeloid Leukemia; CML, Chronic Myeloid Leukemia; CLL, Chronic Lymphocytic Leukemia; HMA, Hypomethylating agent; LSC, Leukemic stem cell; mTOR, Mechanistic target of rapamycin; ROS, Reactive oxygen species; TKI, Tyrosine kinase inhibitor; YY1, Yin-Yang 1.

## 7 Targeting HIF-1α

### 7.1 Advances in nanotherapeutic development

HIF-1α has emerged as a high-priority therapeutic target, driving the development of multiple inhibition strategies. Current approaches focus on four principal mechanisms: HIF1A gene silencing, suppression of HIF-1α protein translation, enhancement of proteasomal degradation, and blockade of HIF-1 transcriptional activity. These modalities collectively aim to attenuate HIF-1α signaling through distinct molecular pathways (150). Nanotechnology has revolutionized HIF-1α-targeted therapy by enabling precise drug delivery systems. Nanotherapeutics leverage unique biological properties to achieve therapeutic monitoring, diagnostic integration, and controlled release kinetics. Engineered nanocarriers with tailored surface modifications and nanoscale dimensions enhance drug delivery selectivity while improving solubility/stability, enabling multi-agent synergism, increasing bioavailability, and facilitating sustained payload release (151). These attributes position nanocarriers as powerful tools for maximizing therapeutic efficacy while minimizing systemic toxicity.

Over recent decades, researchers have engineered diverse nanocarrier architectures including lipid-based nanoparticles, extracellular vesicles (EVs), bioinspired/biohybrid systems, and emerging platforms like metal-organic frameworks (MOFs). This structural diversity not only expands nanomedicine development possibilities but also provides innovative solutions for HIF-1α-targeted interventions (151). Liposomes, as pioneering nanocarriers, have demonstrated clinical success in delivering various HIF-1α-targeted agents (152). EVs, nature-derived nanovesicles, show particular promise for bioactive molecule transport in HIF-1α modulation strategies (153). MOFs offer exceptional drug-loading capacities through their high surface-area-to-volume ratios and programmable porosity, establishing novel platforms for controlled therapeutic release (154).

### 7.2 Nanotherapeutic strategies targeting HIF-1α

#### 7.2.1 HIF-1α siRNA delivery systems

**Figure 3:**
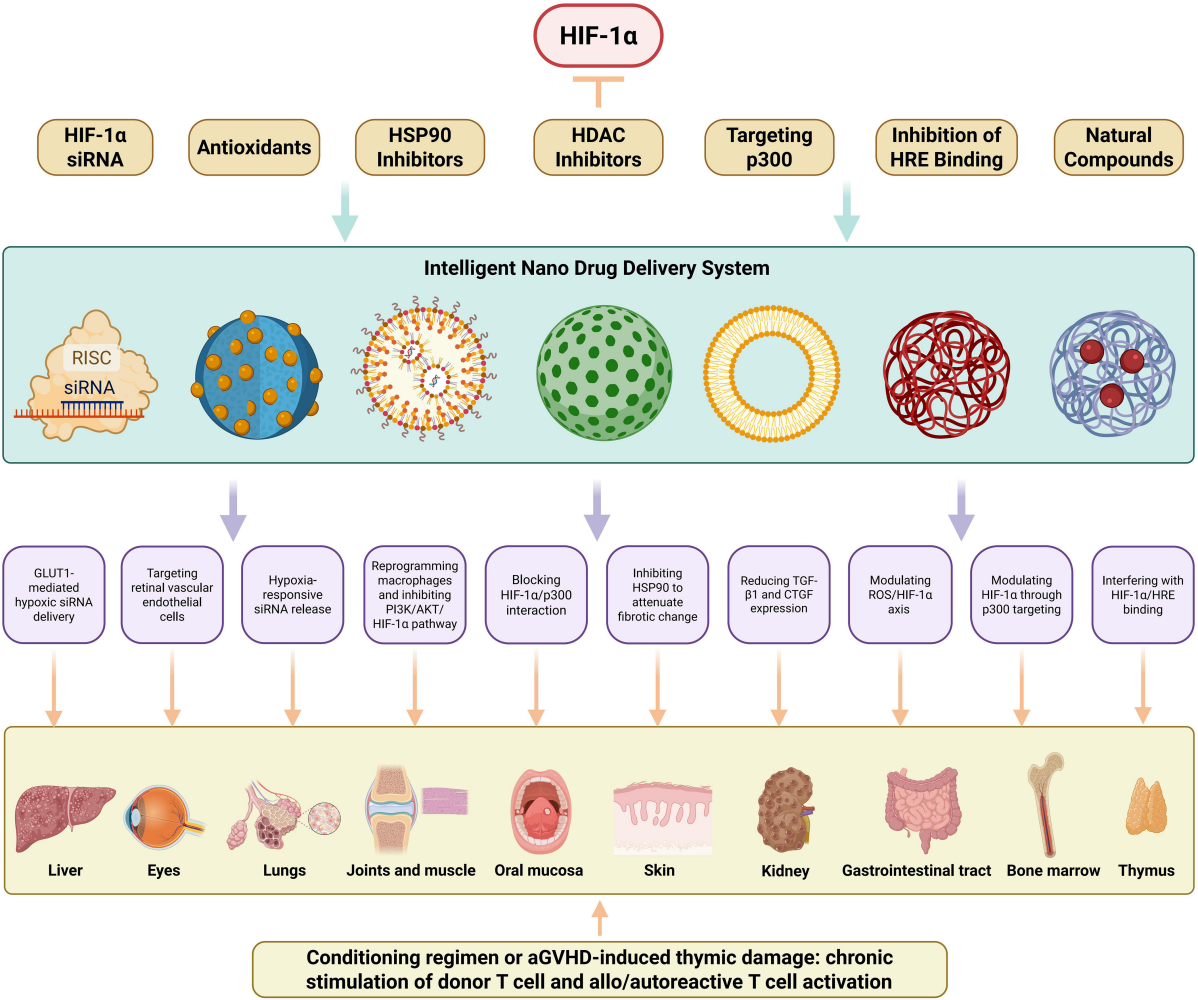
HIF-1α-Targeted Nanotherapeutic Strategies for Organ-Specific cGVHD Intervention. This schematic delineates a multidimensional nanotherapeutic approach targeting HIF-1α in cGVHD. Seven distinct targeting modalities are presented: HIF-1α siRNA delivery, antioxidative nanotherapy, HSP90 inhibition, HDAC inhibition, p300 interference, HRE binding blockade, and phytochemical-based therapy. Collectively, these constitute integrated nano-platforms engineered for HIF-1α gene silencing, enhanced proteasomal degradation, or transcriptional inhibition. An intelligent nanodelivery system-employing diverse nanocarriers (e.g., liposomes, extracellular vesicles, metal-organic frameworks)-facilitates hypoxia-responsive drug release, receptor-mediated targeted delivery, and enhanced biodistribution. This system enables organ-specific precision low-toxicity interventions for cGVHD-affected tissues, including liver (GLUT1-mediated hypoxic siRNA delivery), eyes (Targeting retinal vascular endothelial cells), lungs (Hypoxia-responsive siRNA release), joints/muscles (Reprogramming macrophages and inhibiting PI3K/AKT/HIF-1α pathway), oral mucosa (Blocking HIF-1α/p300 interaction), skin (Inhibiting HSP90 to attenuate fibrotic change), kidneys (Reducing TGF-β1 and CTGF expression), gastrointestinal tract (Modulating ROS/HIF-1α axis), bone marrow (Modulating HIF-1α through p300 targeting), and thymus (Interfering with HIF-1α/HRE binding).

RNA interference-mediated HIF-1α gene silencing represents a potent therapeutic strategy achieved through advanced nanocarrier systems designed to enhance siRNA stability and delivery precision. Cadmium telluride quantum dots functionalized with 2-deoxyglucose enable GLUT1-mediated hypoxic targeting and efficient siRNA delivery through receptor-specific endocytosis mechanisms (155). These nanosystems may target the liver in cGVHD, as the liver is highly metabolically active and glucose transporters like GLUT1 are involved in its normal function and are dysregulated in cGVHD-associated hepatic complications (156). Chimeric membrane-coated iron oxide magnetic nanoparticles demonstrate enhanced tissue penetration capabilities for deep hypoxic zone targeting, as evidenced in recent studies (157). They could potentially target the skin in cGVHD, where deep-seated hypoxic regions may develop due to inflammation and impaired microcirculation, leading to fibrotic changes. Innovative hypoxia-responsive delivery platforms utilizing 2-nitroimidazole-modified polyethyleneimine complexes (bPEI1.8k-C6) achieve oxygen tension-dependent siRNA release through controlled disassembly under low oxygen conditions (158). In cGVHD, these platforms might target the lungs, which are often affected in cGVHD, and the hypoxic microenvironment in the lungs could trigger the release of siRNA to modulate HIF-1α levels (**Figure 3**).

#### 7.2.2 Antioxidant nanotherapeutics

Antioxidant-based nanotherapeutic approaches combat HIF-1α stabilization by maintaining prolyl hydroxylase domain 2 (PHD2) activity through reactive oxygen species (ROS) scavenging. Bilobalide-containing nanoformulations significantly reduce HIF-1α expression in hypoxic cellular environments through enhanced redox homeostasis regulation (159). Ellagic acid encapsulated in nanoliposomal carriers demonstrates dual functionality, simultaneously suppressing HIF-1α activity and protecting against chemotherapy-induced hepatotoxicity in preclinical models (160). Arsenic sulfide mineral-derived nanoparticles exhibit potent ROS/HIF-1α axis modulation, effectively reducing both oxidative stress markers and hypoxia signaling pathways (161). They may target the gastrointestinal tract in cGVHD, where oxidative stress and abnormal HIF-1α activation contribute to mucosal damage and inflammation (162) (**Figure 3**).

#### 7.2.3 HSP90 inhibition platforms

Heat shock protein 90 (HSP90) inhibition strategies leverage nanotechnology to induce proteasomal degradation of HIF-1α through ubiquitination pathways. Nanoporphyrin-based micellar systems co-encapsulating HSP90 inhibitors achieve synergistic HIF-1α suppression through combined photothermal therapy and photodynamic therapy modalities, with the photodynamic component generating cytotoxic singlet oxygen while photothermal effects enhance drug release kinetics (163, 164). These platforms may target the skin in cGVHD, as the skin is easily accessible for photothermal and photodynamic therapies, and modulation of HIF-1α through HSP90 inhibition can potentially reduce fibrotic and inflammatory processes in cGVHD-affected skin (**Figure 3**).

#### 7.2.4 HDAC inhibition strategies

Histone deacetylase (HDAC) inhibitors demonstrate dual therapeutic benefits in both preventing graft-versus-host disease (GVHD) and preserving graft-versus-leukemia (GVL) effects, as evidenced by recent research (165). These compounds additionally promote HIF-1α degradation through epigenetic modulation mechanisms (166). The encapsulation of HDAC inhibitor SAHA within multifunctional photosensitizer-integrated nanogels significantly reduces HIF-1α levels while enhancing photodynamic therapy efficacy (167). The metallofullerenol nanomaterial Gd@C82(OH)22 exhibits multimodal inhibitory activity, functioning as both an HDAC1 inhibitor and a potent suppressor of HIF-1α and TGF-β signaling pathways (168). These may target the lymphoid organs in cGVHD, such as the spleen and lymph nodes, as they play a crucial role in the immune response in cGVHD, and modulation of HIF-1α through HDAC inhibition may help regulate the immune-mediated damage (**Figure 3**).

#### 7.2.5 p300 targeting approaches

The transcriptional coactivator p300 enhances HIF-1α stability through acetylation of its C-terminal domain, amplifying hypoxic signaling under low oxygen conditions (169). Pharmacological intervention using lificiguat (YC-1) disrupts the HIF-1α/p300 interaction, effectively inhibiting transcriptional activation (170). Advanced liposomal systems prepared via film ultrasonic dispersion techniques demonstrate compartmentalized drug loading, with deferoxamine encapsulated in hydrophilic layers and YC-1 in hydrophobic bilayers. This dual-delivery platform maintains iron chelation benefits while preventing HIF-1α overexpression typically induced by deferoxamine (171). The liposomal system may target the bone marrow in cGVHD, as iron metabolism and HIF-1α regulation are important in the bone marrow microenvironment, and modulation of HIF-1α through p300 targeting can potentially improve hematopoiesis and reduce inflammation. Furthermore, the liposomal system encapsulated with YC-1 may also target the kidneys in cGVHD. It has been shown that YC-1 treatment reduces the protein expression levels of TGF-β1 and connective tissue growth factor (CTGF) in diabetic kidneys (172).

Photodynamic therapy challenges are addressed through innovative nanoparticle platforms combining long-lived iridium photosensitizers with YC-1, which mitigate oxygen depletion while suppressing HIF-1α upregulation (173). They could target the ocular surface in cGVHD, where photodynamic therapy can be applied topically, and HIF-1α modulation may alleviate dry eye symptoms and associated inflammation. Although chetomin shows promise in blocking HIF-1α/p300 interactions, its clinical application alongside mTOR inhibitor everolimus is limited by poor solubility-a limitation overcome through mPEG-b-PLA copolymer micelles prepared via solvent evaporation (174). These micelles may target the oral mucosa in cGVHD, as the oral cavity is often affected in cGVHD, and modulation of HIF-1α through p300 targeting can potentially reduce mucosal inflammation and ulceration (**Figure 3**).

#### 7.2.6 HRE binding interference

The formation of active HIF-1 complexes through HIF-1α/β heterodimerization and subsequent binding to hypoxia response elements (HREs) represents a critical therapeutic target. Echinomycin specifically intercalates HRE sequences, effectively blocking transcriptional activation while demonstrating immunomodulatory effects through Tregs cell expansion and Th17/Th1 response reduction, potentially mediated through direct HIF-1α inhibition (175). Nanoliposome-based reformulation of echinomycin enhances therapeutic safety and efficacy through improved pharmacokinetic profiles (152). These nanoliposomal formulations of echinomycin may target the thymus in cGVHD, as the thymus is important for T-cell development and maturation, and modulation of HIF-1α through HRE binding interference can potentially regulate the immune response in cGVHD (**Figure 3**).

#### 7.2.7 Phytochemical-based nanotherapeutics

Natural compounds demonstrate significant potential in HIF-1α modulation through nanotechnology-enhanced delivery systems. Berberine-iron oxide nanoparticle complexes induce proteasome-dependent HIF-1α degradation while enabling magnetic targeting capabilities (176). These complexes may target the heart in cGVHD, as the heart can be affected by cGVHD-related microvascular changes and fibrosis, and magnetic targeting can potentially deliver berberine to the affected cardiac tissues to modulate HIF-1α. Core-satellite structured upconverting nanoparticles loaded with curcumin effectively reprogram macrophages through HIF-1α-mediated photodynamic mechanisms (177). Self-assembling dihydroartemisinin dimer nanoprodrugs (SS NPs) exhibit enhanced stability and glycolytic regulation via PI3K/AKT/HIF-1α pathway inhibition, demonstrating high drug loading capacity (178). These nanoprodrugs may target the joints and muscle tissues in cGVHD and modulation of HIF-1α through glycolytic pathway regulation can potentially improve joints and muscle function (**Table 2**, **Figure 3**).

**Table 2:** Nanotherapeutic strategies targeting HIF-1α.

Targeting strategies	Nanoplatform	Advantages	Limitations	Potential target tissues in cGVHD
HIF-1α siRNA	Cadmium telluride quantum dots functionalized with 2-deoxyglucose (155)	GLUT1-mediated hypoxic targeting, efficient siRNA delivery via receptor-specific endocytosis	Cadmium is potentially toxic, quantum dots may have issues with long-term clearance	Liver
	Chimeric membrane-coated iron oxide magnetic nanoparticles (157)	Enhanced tissue penetration for deep hypoxic zone targeting	Magnetic field application may be required for precise targeting, potential for non-specific magnetic interactions	Skin
	2-nitroimidazole-modified polyethyleneimine complexes (bPEI1.8k-C6) (158)	Oxygen tension-dependent siRNA release	Polyethyleneimine may be cytotoxic at high concentrations	Lungs
Antioxidants	Bilobalide-containing nanoformulations (159)	Reduces HIF-1α expression through redox homeostasis regulation	Uncertainty about long-term effects of bilobalide in nanosystems	Gastrointestinal tract
	Nanoliposomal carriers with ellagic acid (160)	Dual function of suppressing HIF-1α and protecting against hepatotoxicity	Liposomal carriers may have variable stability, potential for immune recognition	Gastrointestinal tract
	Arsenic sulfide mineral-derived nanoparticles (161)	Potent ROS/HIF-1α axis modulation	Arsenic may have toxicity concerns, nanoparticles may aggregate	Gastrointestinal tract
HSP90 Inhibitors	Nanoporphyrin-based micellar systems co-encapsulating HSP90 inhibitors (163, 164)	Synergistic HIF-1α suppression via combined photothermal and photodynamic therapies, enhanced drug release with photothermal effect	Phototherapy may require specific light sources, potential for tissue damage from over-exposure	Skin
HDAC Inhibitors	Multifunctional photosensitizer-integrated nanogels with SAHA (167)	Reduces HIF-1α levels, enhances photodynamic therapy efficacy	Nanogel stability may be affected by physiological conditions, potential for immune response to nanogels	Lymphoid organs (spleen, lymph nodes)
	Metallofullerenol nanomaterial Gd@C82(OH)22 (168)	Multimodal inhibitory activity on HDAC1, HIF-1α, and TGF-β	Metallofullerenol may have issues with solubility and clearance	Lymphoid organs (spleen, lymph nodes)
Targeting p300	Liposomal systems with deferoxamine and YC-1 (171)	Compartmentalized drug loading, prevents HIF-1α overexpression by deferoxamine	Liposome stability may vary, potential for leakage of encapsulated drugs	Bone marrow/Kidney
	Nanoparticle platforms with long-lived iridium photosensitizers and YC-1 (173)	Mitigates oxygen depletion, suppresses HIF-1α upregulation	Photodynamic therapy may have limited tissue penetration, potential for photosensitizer-related side effects	Ocular surface
	mPEG-b-PLA copolymer micelles with chetomin and everolimus (174)	Overcomes solubility issue of chetomin	Micelle formation may be complex, potential for variable drug release	Oral mucosa
Inhibition of HRE Binding	Nanoliposome-based echinomycin (152, 175)	Enhanced therapeutic safety and efficacy, improved pharmacokinetic profiles	Liposomal carriers may be cleared relatively quickly, potential for immune recognition	Thymus
Natural Compounds	Berberine-iron oxide nanoparticle complexes (176)	Induces proteasome-dependent HIF-1α degradation, magnetic targeting	Iron oxide nanoparticles may cause magnetic interference in some imaging modalities, potential for berberine-related side effects	Heart
	Core-satellite structured upconverting nanoparticles with curcumin (177)	Reprograms macrophages through HIF-1α-mediated photodynamic mechanisms	Upconverting nanoparticles may have complex synthesis procedures, photodynamic therapy may have limited tissue penetration	Joints and muscle tissues
	Self-assembling dihydroartemisinin dimer nanoprodrugs (SS NPs) (178)	Enhanced stability, glycolytic regulation via PI3K/AKT/HIF-1α pathway inhibition, high drug loading capacity	Nanoprodrug activation and release may be affected by physiological conditions	Joints and muscle tissues

### 7.3 Addressing practical challenges in nanotherapeutic translation​

While the preceding nanotherapeutic strategies targeting HIF-1α demonstrate considerable preclinical promise for mitigating cGVHD manifestations in specific organs, their successful clinical translation necessitates a rigorous assessment and proactive mitigation of inherent practical challenges.

#### 7.3.1 Scalability in production

A primary translational hurdle involves achieving scalable and reproducible manufacturing of complex nanocarriers. The intricate architectures, specialized surface functionalizations, and precise drug loading requirements mandate sophisticated production processes susceptible to batch-to-batch variability, posing significant barriers to cost-effective, large-scale Good Manufacturing Practice (GMP) compliant production required for clinical trials and commercialization (179). Mitigation strategies center on adopting modular nanocarrier designs amenable to scalable production methods, implementing Quality-by-Design (QbD) principles for robust process optimization and control, and leveraging advanced analytical techniques for exhaustive physicochemical characterization to ensure batch consistency (180).

#### 7.3.2 Regulatory hurdles and compliance strategies

Navigating the evolving regulatory landscape for nanomedicines presents distinct complexities. Regulatory agencies face the challenge of evaluating novel material interactions, complex structure-activity relationships, potential long-term biodistribution profiles, and unique safety profiles that may differ significantly from traditional small molecules or biologics. Pre-emptive mitigation involves early and continuous engagement with regulatory bodies to define appropriate non-clinical testing pathways, developing comprehensive safety assessment programs that include extensive *in vitro* immunogenicity screening and specialized *in vivo* toxicology studies evaluating organ accumulation and chronic effects, and establishing standardized characterization protocols for novel nanoplatforms (181).

#### 7.3.3 Pharmacokinetics, toxicity, and optimization strategies

The pharmacokinetic profile and potential toxicity of nanotherapeutics remain critical considerations. The modifications that confer enhanced permeability and retention (EPR) or active targeting capabilities can also lead to unintended interactions with the mononuclear phagocyte system (MPS), resulting in accelerated blood clearance, significant liver/spleen sequestration, and potential off-target toxicities upon repeated dosing, thereby limiting bioavailability at the intended pathological site and increasing systemic burden (182). Moreover, the biodistribution and long-term biocompatibility of both the nanocarrier components and their degradation products must be thoroughly investigated (183). Mitigation approaches for pharmacokinetic and toxicity challenges focus on strategic material selection, surface engineering to minimize MPS recognition, rational dose optimization guided by detailed pharmacokinetic/pharmacodynamic (PK/PD) modelling in relevant disease models, and the development of predictive *in vitro* and in silico models to better estimate human safety margins.

#### 7.3.4 Biological barriers and translational strategies

While HIF-1α-targeted nanotherapeutics show preclinical promise for cGVHD, clinical translation requires addressing disease-specific biological hurdles. Key challenges include aligning nanocarrier design with cGVHD’s multifocal nature (navigating vascular dysfunction and avoiding MPS clearance), resolving HIF-1α’s dual role (balancing fibrosis inhibition with tissue repair), and developing hypoxia-responsive release systems. Preclinical models must recapitulate cGVHD’s immunological complexity, while biomarker discovery should enable non-invasive efficacy monitoring (184). Regulatory strategies should leverage adaptive trials for organ-specific evaluation, integrating nanomedicine design with cGVHD pathophysiology to restore immune tolerance and tissue homeostasis.

## 8 Discussion and outlook

As a potential therapeutic target for cGVHD, HIF-1α has garnered attention due to its multifaceted roles in immune regulation, metabolic reprogramming, and fibrosis. Inhibiting HIF-1α can simultaneously intervene in inflammatory responses, abnormal T/B cell activation, and tissue fibrosis, demonstrating the advantage of “one target, multiple effects.” For example, using nanocarriers to deliver HIF-1α siRNA or small molecule inhibitors (such as Echinomycin) can precisely downregulate its expression, alleviating Th17 polarization and the fibrotic process; regulating the gut microbiota-HIF-1α axis may restore immune homeostasis, providing new ideas for combination therapy. However, there are still many challenges that cannot be ignored. The functions of HIF-1α in normal physiology (such as hypoxic adaptation) may lead to tissue damage if overly suppressed; the *in vivo* stability of nanomedicines and their ability to penetrate fibrotic tissues still need optimization; some inhibitors (such as HDAC inhibitors) may interfere with epigenetic regulation, leading to off-target effects; moreover, most studies are limited to animal models, requiring further validation of their safety and long-term efficacy. Future developments could include smart responsive nanocarriers (such as hypoxia-triggered drug release), combined targeting of HIF-1α with other pathways (such as PD-1/CTLA-4) (185, 186), and utilizing organoid models to screen for efficient and low-toxicity compounds. Despite the challenges, HIF-1α-targeted therapy shows great promise in cGVHD, and integrating precision medicine with nanotechnology may open new therapeutic paradigms for this refractory disease.
